# Improvements in small airway dysfunction and respiratory symptoms following tezepelumab for severe asthma

**DOI:** 10.1183/23120541.01593-2025

**Published:** 2026-07-20

**Authors:** Aviv Kupershmidt, Gal Elkayam, Nimrod Urtreger, Ariel Malloul, Inbal Regev-Friedman, Avraham Unterman, Shai Moshe Amor, Sharon Enghelberg, Ophir Freund, Amir Bar-Shai

**Affiliations:** Institute of Pulmonology, Tel Aviv Sourasky Medical Center, Tel Aviv University, Tel Aviv, Israel

## Abstract

**Background:**

Small airway dysfunction (SAD) is a key feature of severe asthma, contributing to poor symptom control and exacerbations. Tezepelumab has demonstrated efficacy in reducing exacerbations and improving lung function, but its specific effects on SAD remain underexplored. We aimed to assess changes in SAD among patients initiating tezepelumab.

**Methods:**

A prospective observational study was carried out among patients with severe asthma treated with tezepelumab in a tertiary centre. SAD was assessed at baseline and 6 months using impulse oscillometry (IOS) and spirometry. We focused on frequency dependence of resistance (*R*_5–20_) and the area under the reactance curve (AX) to define SAD, with pathological cut-offs >0.1 kPa·L^−1^·s^−1^ and >1.0 kPa·L^−1^, respectively. Paired analyses were used to compare changes over time.

**Results:**

34 patients were included (median age 60 years, 74% females, 21% biologic-experienced) with a median follow-up of 183 days. There were significant improvements from baseline to follow-up in forced expiratory volume in 1 s (FEV_1_) (median 1.73 *versus* 1.97 L, p=0.001), mid-expiratory flow at 25–75% of forced vital capacity (FVC) (median 1.26 *versus* 1.73 L·s^−1^, p=0.002), *R*_5−20_ (median 0.16 *versus* 0.08 kPa·L^−1^·s^−1^, p<0.001) and AX (median 1.62 *versus* 0.80 kPa·L^−1^, p<0.001). Improvements in IOS parameters were larger in patients with increased Asthma Control Test (ACT) scores ≥3 points. Prevalence of SAD decreased from 76% to 44% at follow-up (p=0.003). SAD at follow-up was associated with smoking, lower FEV_1_ and FVC, and smaller improvements in ACT score.

**Conclusion:**

Tezepelumab significantly improved small airway function, which was associated with better asthma control. Our findings highlight SAD as a treatable trait in patients with severe asthma.

## Shareable abstract

**Tezepelumab treatment was associated with meaningful improvement in small airway function and better asthma control in a real-world cohort. Small airway function is an important treatable trait in severe asthma.** https://bit.ly/4rlbuhH

## Introduction

Small airway dysfunction (SAD) plays a pivotal role in the pathophysiology of asthma and has been increasingly recognised as a key determinant of poor disease control. The small airways, defined as those with an internal diameter of <2 mm, contribute substantially to airflow obstruction, inflammation and remodelling [1, 2]. Because conventional spirometry is relatively insensitive to changes in the distal airways, SAD may remain undetected even in patients with normal spirometric results [3]. On the other hand, impulse oscillometry (IOS), a noninvasive and effort-independent technique, has emerged as a sensitive tool to detect small airway involvement [4, 5]. Accumulating evidence links SAD with worse asthma outcomes, including increased symptoms, airway hyperresponsiveness, accelerated lung function decline and higher risk of exacerbations [1, 6]. This highlights the importance of targeted therapeutic strategies, aiming to improve distal airway function and enhance overall disease management [1, 7].

A growing body of evidence indicates that biologic therapies can influence SAD, with considerable variability in study design and reported outcomes. Anti-interleukin (IL)-5 agents, such as mepolizumab and benralizumab, have shown heterogeneous effects in some reports [8–10]. However, more recent prospective and clinical data support a beneficial effect on oscillometry-derived indices of distal airway function [11, 12]. Therapies targeting the IL-4/IL-13 pathway have also demonstrated favourable effects on small airway mechanics in several recent studies [13–16]. Tezepelumab, an anti-thymic stromal lymphopoietin (anti-TSLP) therapy, has been previously shown to reduce asthma exacerbations and improve symptoms in multiple randomised controlled trials and multicentre real-world studies [17, 18]. Considering tezepelumab's broad upstream anti-inflammatory action, it might also have a positive effect on SAD [19], and indeed several recent studies have reported improvements in oscillometry-derived parameters following treatment, although the available evidence is currently based on small exploratory cohorts [20–22].

Based on the above, we aimed to evaluate the change in SAD following tezepelumab initiation in patients with severe asthma, predictors for its improvement and associations with other disease outcomes. We hypothesised that treatment with tezepelumab would lead to measurable improvement in IOS-derived parameters reflecting distal airway function.

## Methods

### Study design

This was a prospective cohort study including consecutive patients that initiated tezepelumab for severe asthma at a large tertiary medical centre. All patients initiating tezepelumab were eligible and invited to participate in this study. Participants completed clinical and physiological assessments at baseline (up to 2 h prior to first tezepelumab dose) and at a 6-month follow-up visit. Patients were excluded if they had <4 months of follow-up or were unable to perform one of the study evaluations. None of the patients met any of the exclusion criteria. Tezepelumab was administered per routine clinical practice, *i.e.* 210 mg every 4 weeks for severe asthma.

Patients were approached for participation during their visit and all signed an informed consent. The study was approved by our institutional review board (TLV-0106-24) and adheres to the Declaration of Helsinki.

### Patients’ data and data analysis

Patients’ data were collected from a case report form completed by patients at their first treatment, and validated by reviewing all electronic medical records. Data were collected and entered into a standardised database. Baseline data refer to data prior to treatment initiation and included patient demographics, exacerbation rate and maintenance treatment, eosinophil levels (highest in prior 6 months), pulmonary function tests and IOS results. Exacerbation rates were calculated as 6-month exacerbations rate (6mER), with the following formula: (total exacerbations/total days of follow-up) ×180. Exacerbations were defined as worsening in respiratory symptoms that have led to oral corticosteroid treatment.

The cohort was divided into three groups, based on prior real-world studies [17]: 1) prior biologics experienced – patients who received a biologic therapy for severe asthma during the pre-treatment period; 2) naïve with peripheral eosinophil count ≥150 cells·μL^−1^; and 3) naïve with peripheral eosinophil count of <150 cells·μL^−1^. Of note, all prior biologic therapies were taken for at least 6 months before switching to tezepelumab. In all cases, patients switched their biologic therapy based on lack of control in symptoms or exacerbations. Given the real-world design, there was no washout period between treatments.

As part of routine follow-up in patients receiving biologics, spirometry and IOS are performed without bronchodilators; therefore, only pre-bronchodilator values were included. Spirometry indices included forced expiratory volume in 1 s (FEV_1_, L), forced vital capacity (FVC, L) and mid-expiratory flow at 25–75% of FVC (MEF_25–75_, L·s^−1^). IOS parameters included total airway resistance at 5 Hz (*R*_5_, kPa·L^−1^·s^−1^), central airway resistance at 20 Hz (*R*_20_, kPa·L^−1^·s^−1^), the difference between *R*_5_ and *R*_20_ (*R*_5−20_, kPa·L^−1^·s^−1^) and area under the reactance curve (AX, kPa·L^−1^). IOS tests were performed with the patient sitting and with nose clips. Patients performed tidal breathing for 30 s while firmly supporting their cheeks. The procedure was repeated three times to ensure accuracy and reproducibility, with each test being reviewed for artefacts. All tests were conducted in accordance to accepted guidelines [23–25], with daily calibration and by an experienced respiratory technician. In addition, asthma control was evaluated using the Asthma Control Test (ACT), a validated patient-reported questionnaire assessing symptom control over the previous 4 weeks [26].

### Data analysis

*R*_5−20_ and AX values were assessed both as continuous variables and using a validated cut-off to define SAD of 0.1 kPa·L^−1^·s^−1^ and 1.0 kPa·L^−1^, respectively [27–29]. Continuous variables are presented as median (interquartile range (IQR)) and categorical variables as sum (percentage from total). Comparisons of continuous variables from baseline to follow-up were performed by related-samples Wilcoxon signed rank tests. Differences in continuous variables between groups were assessed using Mann–Whitney U-tests (for two groups) or Kruskal–Wallis tests (>two groups). Categorical variables were compared from pre- to post-procedure using McNemar's tests for paired analysis. All analyses were performed in SPSS version 30.0.

## Results

During the study period, 34 patients with severe asthma initiated tezepelumab and completed the 6-month follow-up (median 183 days (153–210)). The cohort characteristics are shown in table 1. Median (IQR) age was 60 years (48–70), 74% were female and 9% were on mOCS. Overall, 21% had prior biologic treatment (biologic experienced), and of those naïve to biologics, 44% had eosinophil levels below 150 cells·µL^−1^. Overall, eight patients (24%) were treated with an extra-fine particle inhaler and 19 (56%) with triple therapy (single or multiple inhalers), and there were no changes in the inhaler type during the study period. Prior biologic therapy included dupilumab (n=3), benralizumab (n=2) and mepolizumab (n=2).

**TABLE 1:** Study cohort characteristics (n=34)

**Age years**	60 (48–70)
**Female sex**	25 (74)
**Smoking**	
Never	26 (77)
Prior	5 (15)
Current	3 (9)
**Obese (BMI >30** **kg·m^−2^)**	4 (12)
**Allergy**	17 (50)
**CRS-NP**	3 (9)
**Bronchiectasis**	4 (12)
**COPD**	2 (6)
**Cardiovascular risk factors**	8 (24)
**6** **months highest eosinophils cells·µL^−1^**	100 (100–200)
**Inhalers**	
Extra-fine particle inhaler	8 (24)
Triple-inhaler therapy^#^	19 (56)
**Maintenance OCS**	3 (9)
**Group**	
Naïve, eosinophils ≥150 cells·µL^−1^	12 (35)
Naïve, eosinophils <150 cells·µL^−1^	15 (44)
Biologic experienced	7 (21)

Data are presented as median (IQR) or n (%). BMI: body mass index; CRS-NP: chronic rhinosinusitis with nasal polyposis; OCS: oral corticosteroids. ^#^: includes long-acting muscarinic antagonist, β-agonist and inhaled corticosteroids, either in a single inhaler or multiple.

**FIGURE 1:**
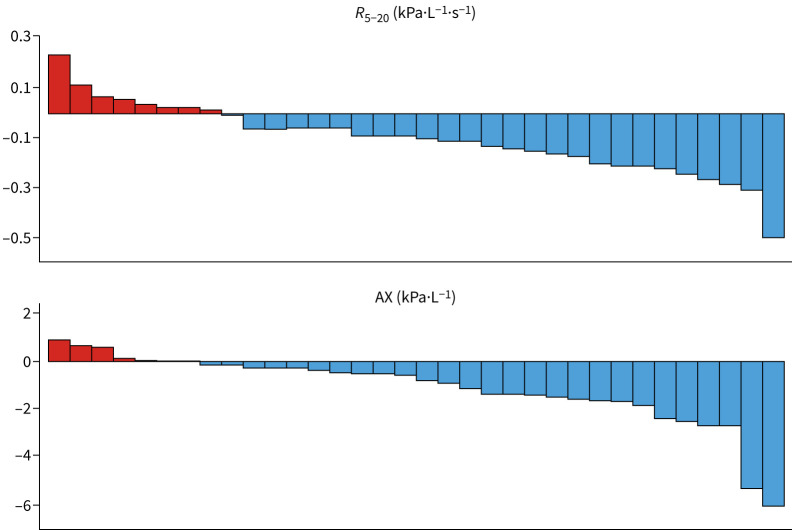
Changes in *R*_5−20_ (upper panel) and AX (lower panel) from baseline to follow-up. Each column represents a patient, with blue for reduction in values (improvement) and red for an increase (worsening) over time. AX: area of reactance; *R*_5/20_: resistance at 5/20 Hz.

Changes in lung function, ACT scores and exacerbations from baseline to follow-up are shown in table 2. At follow-up, significant improvements were found in forced expiratory volume in 1 s (FEV_1_) (median (IQR) 1.73 (1.18–2.26) *versus* 1.97 (1.36–2.34) L) and MEF_25–75_ (1.26 (0.57–2.13) *versus* 1.73 (0.80–2.54) L·s^−1^). Changes were also found for IOS small airway parameters (figure 1), including *R*_5–20_ (from 0.16 (0.09–0.26) to 0.08 (0.04–0.12) kPa·L^−1^·s^−1^, p<0.001), and AX (from 1.62 (0.73–2.86) to 0.80 (0.32–1.37) kPa·L^−1^, p<0.001). In addition, ACT scores improved from a median (IQR) of 16 (11–20) to 19 (16–23). 6mER reduced from a mean ±sd of 1.79±0.8 to 0.39±0.6, representing a 78% reduction in exacerbations.

**TABLE 2:** Comparison of asthma-related parameters from baseline to follow-up

Variable	Baseline	Follow-up	Mean difference (95% CI)	p-value^#^
**FEV_1_, L**	1.73 (1.18–2.26)	1.97 (1.36–2.34)	0.24 (−0.19–1.08)	0.001
**FEV_1_, % pred**	72 (47–89)	79 (57–96)	6.7 (−12–26)	0.018
**FVC, L**	2.63 (2.20–3.0.7)	2.71 (2.41–3.09)	0.09 (−0.38–0.53)	0.072
**FVC, % pred**	84 (71–100)	89 (78–101)	3.3 (−17–24)	0.190
**MEF_25–75_, L**	1.26 (0.57–2.13)	1.73 (0.80–2.54)	0.24 (−0.57–0.88)	0.002
**MEF_25–75_, % pred**	35 (19–58)	53 (28–78)	14 (−17–31)	0.002
***R*_5_, kPa·L^−1^·s^−1^**	0.52 (0.45–0.65)	0.41 (0.39–0.45)	0.14 (−0.14–0.43)	<0.001
***R*_20_, kPa·L^−1^·s^−1^**	0.37 (0.32–0.45)	0.34 (0.31–0.38)	0.04 (−0.7–0.17)	0.022
***R*_5−20_, kPa·L^−1^·s^−1^**	0.16 (0.09–0.26)	0.08 (0.04–0.12)	0.10 (−0.11–0.30)	<0.001
**AX, kPa·L^−1^**	1.62 (0.73–2.86)	0.80 (0.32–1.37)	1.10 (−0.72–5.26)	<0.001
**ACT score**	16 (11–20)	19 (16–23)	3 (−2–9)	<0.001
**6mER**	1.7 (1.04–2.20)	0 (0–0.9)	1.42 (0.98–1.97)	<0.001

Data are presented as median (IQR), unless indicated otherwise. FEV_1_: forced expiratory volume in 1 s; FVC: forced vital capacity; MEF_25–75_: mid-expiratory flow at 25–75% of FVC; *R*_5/20_: resistance at 5/20 Hz; AX: area of reactance; ACT: Asthma Control Test; 6mER: 6 months exacerbation rate. ^#^: comparisons were made using paired analyses.

**FIGURE 2:**
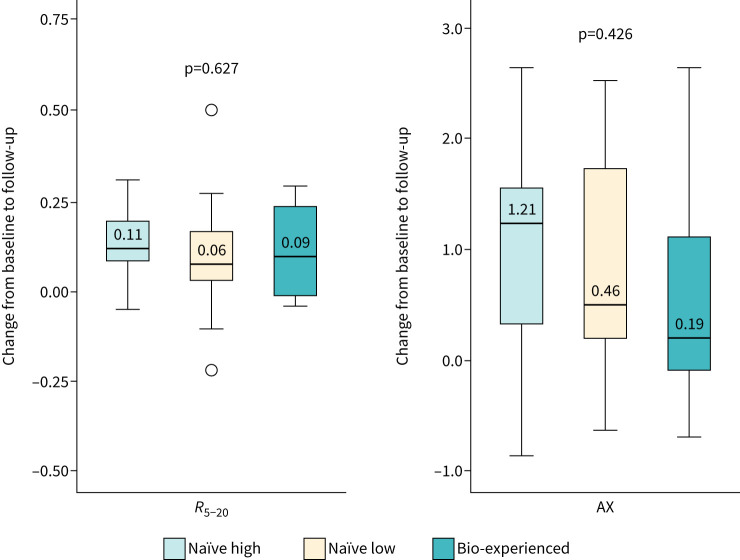
Changes in *R*_5−20_ and AX from baseline to follow-up between the study groups. The naïve high group refers to patients naïve to biologic therapy with eosinophil counts of ≥150 cells·µL^−1^. AX: area of reactance; *R*_5/20_: resistance at 5/20 Hz.

**FIGURE 3:**
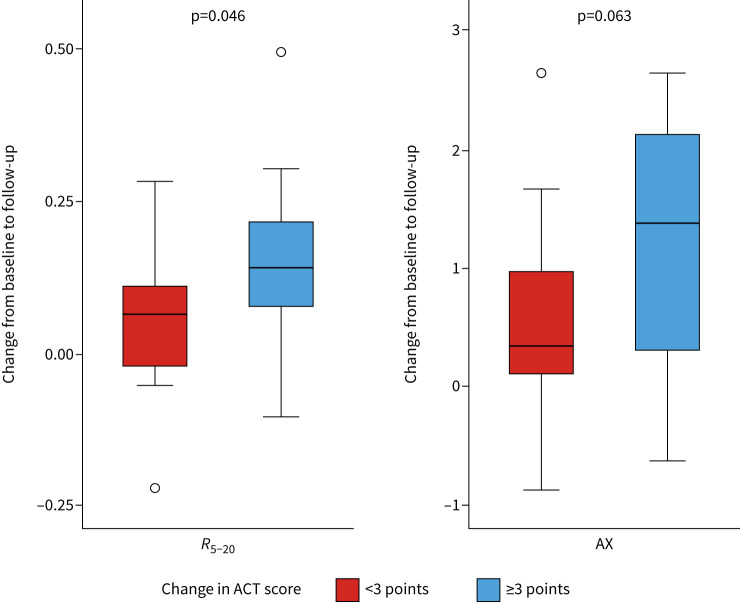
Changes in *R*_5−20_ and AX from baseline to follow-up stratified by improvements of Asthma Control Test (ACT) score above and below 3 points. AX: area of reactance; *R*_5/20_: resistance at 5/20 Hz.

Changes in *R*_5–20_ and AX were not associated with baseline patient characteristics, including whether patients were using an extra-fine particle inhaler. Similar improvements were also found between the biologic experienced and biologic-naïve patients with low or high eosinophils (figure 2). Half of the cohort (n=17) had a decrease in AX of above 0.65 kPa·L^−1^, the minimal clinically important difference (MCID) for this variable [30]. Patients with increased ACT scores of 3 or more (the MCID for ACT [31]) at follow-up also had larger improvements in *R*_5–20_ (figure 3, median decrease of 0.14 *versus* 0.06 kPa·L^−1^·s^−1^) and AX (median decrease of 1.35 *versus* 0.30 kPa·L^−1^).

### Small airway dysfunction

**FIGURE 4:**
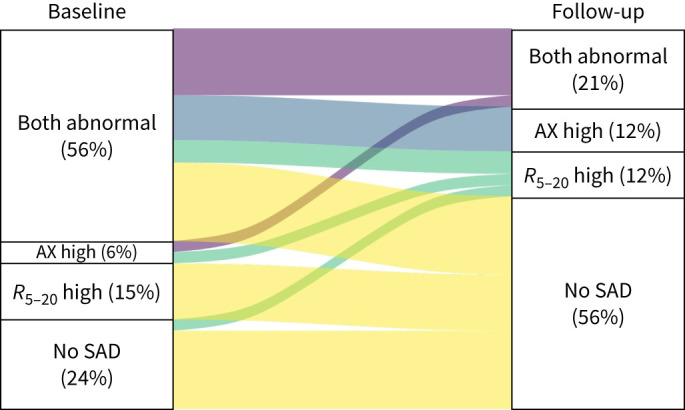
Changes in small airway dysfunction (SAD) of *R*_5−20_, AX, or both from baseline to follow-up. AX: area of reactance; *R*_5/20_: resistance at 5/20 Hz.

SAD was defined by *R*_5–20_ >0.1 kPa·L^−1^·S^−1^ or AX >1.0 kPa·L^−1^, as described in the Methods. At baseline, 24% had no SAD, while 56% had both abnormal *R*_5–20_ and AX. There were significant changes at follow-up (figure 4), with only 21% having both abnormal indices and 56% without SAD (p=0.003)

Next, we compared patient characteristics between those with and without SAD at follow-up (table 3). SAD at follow-up was more prevalent in patients with prior or current smoking (47% *versus* 5%, OR 15.8, 95% CI 1.65–150, p=0.013), and those with lower MEF_25–75_ at baseline (OR 0.49, 95% CI 0.23–0.96) and FVC at baseline (OR 0.45, 95% CI 0.20–0.94). In addition, follow-up SAD was associated with a lower FEV_1_ at follow-up (median (IQR) 1.49 (1.1–2.0) *versus* 2.27 (1.8–2.5) L, OR 0.34, 95% CI 0.12–0.98, p=0.040) and reduced improvements in ACT score (median (IQR) increase of 1 (0–3) *versus* 3 (2–7), OR 0.68, 95% CI 0.49–0.94, p=0.033).

**TABLE 3:** Comparison of characteristics and outcomes between patients with and without small airway dysfunction at follow-up

Variable	Follow-up SAD	No SAD	p-value
**Patients, n**	15	19	
**Age, years**	68 (42–72)	56 (50–70)	0.471
**Female sex**	12 (80)	13 (68)	0.448
**Prior/current smoking**	7 (47)	1 (5)	0.013
**Allergy**	7 (47)	10 (53)	0.730
**Bronchiectasis**	3 (20)	1 (5)	0.185
**Biologic naïve, eosinophils ≥150** **cells·µL**	6 (40)	6 (32)	0.610
**Baseline FEV_1_, L**	1.33 (0.6–1.9)	2.05 (1.3–2.4)	0.062
**Baseline FVC, L**	2.41 (1.7–2.9)	2.95 (2.5–3.5)	0.043
**Baseline MEF_25–75_, L**	0.70 (0.4–1.5)	1.63 (0.8–2.7)	0.026
**Follow-up FEV_1_, L**	1.49 (1.1–2.0)	2.27 (1.8–2.5)	0.041
**Follow-up FVC, L**	2.53 (1.7–2.9)	2.77 (2.5–3.8)	0.066
**Follow-up MEF_25–75_, L**	0.86 (0.6–2.3)	2.05 (1.6–2.7)	0.071
**Change in ACT from baseline**	1 (0–3)	3 (2–7)	0.033

Data are presented as median (IQR) or n (%). SAD: small airway dysfunction; FEV_1_: forced expiratory volume in 1 s; FVC: forced vital capacity; MEF_25–75_: mid-expiratory flow at 25–75% of FVC; ACT: Asthma Control Test.

## Discussion

In this prospective real-world study, we evaluated the effect of tezepelumab on SAD in patients with severe asthma. SAD was assessed with IOS given its simplicity to perform, lack of dependence on operator or patient performance, and high sensitivity [32]. The main strength of our study lies in its prospective real-world design, which reflects routine clinical practice and includes a heterogeneous patient population. Treatment with tezepelumab was associated with significant improvement in SAD, and this physiological improvement correlated with enhanced asthma control and improvement in other physiological indices. These results suggest that tezepelumab may have a potential beneficial effect on distal airway physiology, accompanied by measurable clinical improvement.

The improvement in small airway function observed in our cohort may reflect the unique upstream mechanism of tezepelumab. By blocking TSLP, tezepelumab inhibits multiple downstream inflammatory pathways, including both type 2 (IL-4, IL-5, IL-13) and non-type 2 mediators, resulting in broader suppression of airway inflammation and remodelling [19, 33]. Supporting this concept, a mechanistic *ex vivo* study in human small airway tissue by Manson *et al*. [34] demonstrated that IL-4/IL-13 signalling is a key driver of small airway hyperresponsiveness, whereas IL-5-mediated pathways were not, providing direct tissue-level evidence for differential cytokine effect. In addition, a recent study by Chan *et al*. [35] compared the effects of dupilumab and benralizumab on small airways using oscillometry and demonstrated significantly greater improvements in peripheral resistance and reactance with dupilumab, likely due to its inhibition of IL-13 signalling. Collectively, these findings suggest that biologics acting upstream of IL-13, such as tezepelumab, may have the potential to exert a more pronounced effect on small airways than agents acting downstream on IL-5 pathways, such as benralizumab.

The strong association between changes in IOS parameters and ACT score highlights the clinical relevance of small airway function in determining overall asthma control. IOS reflects mechanical abnormalities that may not be captured by spirometry, and improvements in resistance and reactance are likely to correlate with symptomatic relief such as reduced dyspnoea, cough and wheezing [36]. This finding supports the growing body of evidence linking physiological improvement in the distal airways to enhanced quality of life and better symptom perception [37, 38]. Our results suggest that monitoring SAD through oscillometry may provide complementary information to patient-reported outcomes and may serve as a sensitive indicator of treatment response to biologic therapy. Moreover, IOS may be considered as part of the asthma's biomarker arsenal, with its results taken into consideration prior to biologic therapy selection.

When comparing patient characteristics between those who showed resolution of SAD and those in whom it persisted at follow-up, we observed that persistent SAD was more common among individuals with a history of smoking and in those presenting with lower baseline lung function parameters. Previous studies have shown that reduced baseline lung function is associated with a poorer response to asthma treatment [39, 40]. Our results suggest that this relationship extends to the small airways, indicating that patients with more advanced physiological impairment at baseline are less likely to experience improvement in IOS-derived indices. Similarly, smoking has previously been shown to contribute to airway remodeling and irreversible structural changes [41, 42], and our findings highlight the relevance of these processes also within the small airways compartment. Higher body mass index was previously shown to correlate with SAD and might also be a predictor for lowered treatment effect [43, 44]. This should be assessed by future larger studies, considering that our sample size and low rate of obese patients was underpowered to allow further analyses. Finally, the inhaler type might also be a mitigating factor in SAD improvement over time. Although we did not find such association in our cohort, studies have shown better lung distribution of inhaled extra-fine therapy [45], though prospective controlled studies are needed to support their superiority for small airway disease.

This study has several limitations that should be acknowledged. Most importantly, the observational real-world design lacked a control group, limiting causal inference regarding the effect of tezepelumab on small airway function. In the absence of a comparator group, the magnitude of the observed effect cannot be precisely quantified and spontaneous disease variability cannot be excluded. Second, the sample size was relatively small, and although this is the largest cohort to date on this topic, larger cohorts would allow more robust subgroup analyses. Third, adherence with inhaler therapy was not assessed and might also affect the change in SAD at follow-up. Fourth, exhaled nitric oxide fraction testing was unavailable in our centre for most of the study period, limiting the full characterisation of our cohort. Finally, the follow-up duration was limited to the early treatment period, and longer-term studies are required to assess the persistence of the observed improvements.

In conclusion, tezepelumab treatment was associated with improvement in small airway function after 6 months of therapy. These changes were associated with symptomatic and other physiological improvements. As a real-world study, its results reflect treatment outcomes in a diverse patient population, supporting the potential generalisability of tezepelumab's effect in severe asthma. Our findings support the concept of SAD as a treatable trait as well as a potential biomarker in severe asthma and suggest that targeting upstream inflammatory pathways may lead to meaningful physiological and clinical benefits.

## Data Availability

All relevant data and analyses are given within the manuscript.
